# Predation risk landscape modifies flying and red squirrel nest site occupancy independently of habitat amount

**DOI:** 10.1371/journal.pone.0194624

**Published:** 2018-03-29

**Authors:** Tytti Turkia, Erkki Korpimäki, Alexandre Villers, Vesa Selonen

**Affiliations:** 1 Section of Ecology, Department Biology, University of Turku, Turku, Finland; 2 French National Centre for Agricultural Research, Chizé, France; Université de Sherbrooke, CANADA

## Abstract

Habitat choice often entails trade-offs between food availability and predation risk. Understanding the distribution of individuals in space thus requires that both habitat characteristics and predation risk are considered simultaneously. Here, we studied the nest box use of two arboreal squirrels who share preferred habitat with their main predators. Nocturnal Ural owls (*Strix uralensis*) decreased occurrence of night-active flying squirrels (*Pteromys volans*) and diurnal goshawks (*Accipiter gentilis*) that of day-active red squirrels (*Sciurus vulgaris*). Unexpectedly, the amount of preferred habitat had no effect on nest box use, but, surprisingly, both squirrel species seemed to benefit from close proximity to agricultural fields and red squirrels to urban areas. We found no evidence of trade-off between settling in a high-quality habitat and avoiding predators. However, the amount of poor-quality young pine forests was lower in occupied sites where goshawks were present, possibly indicating habitat specific predation on red squirrels. The results suggest that erecting nest boxes for Ural owls should be avoided in the vicinity of flying squirrel territories in order to conserve the near threatened flying squirrels. Our results also suggest that flying squirrels do not always need continuous old forests, and hence the currently insufficient conservation practices could be improved with reasonable increases in the areas left untouched around their nests. The results of this study demonstrate the importance of taking into account both habitat requirements and predation risk as well as their interactive effects when modeling the occupancy of threatened animal species and planning their conservation.

## Introduction

When choosing a habitat, animals face trade-offs between food availability, refuges against predators, parasite avoidance, and other specific habitat characteristics. Natural selection favors optimization of these effects on fitness in different habitats [1]. For example, predators have habitat specific effects on prey directly by inducing mortality (e.g. [2]), but also indirectly through creating what is called a predation risk landscape [3], or a landscape of fear [4, 5]. The indirect effects of predators may affect movement and habitat use of prey individuals, and ultimately, the whole population [6, 7]. Therefore, individuals of prey species are more likely to occupy sites with low risk of encountering a predator [8]. Together, habitat fragmentation and predation pressure may modify population densities more or in a different manner than either factor alone [9–11]. This highlights the importance of considering both factors when aiming to understand the distribution of animals in space [12]. However, earlier studies on simultaneous effects of habitat composition and predation risk on species occurrence have focused mainly on certain taxa, like fish [13, 14], birds [15–17] and voles and mice [18, 19], whereas other mammals remain little studied.

Arboreal squirrels have an important role in many forest communities being, for example, important prey for several avian predators [20–24]. Furthermore, their main habitat is often under heavy human use, which makes them potentially sensitive to habitat composition at both patch and landscape level [25, 26]. For example, the Siberian flying squirrel (*Pteromys volans*, hereafter flying squirrel) and the Eurasian red squirrel (*Sciurus vulgaris*, hereafter red squirrel) are declining species living in boreal forests, both potentially suffering from habitat loss in managed forest landscape [27, 28]. However, relative roles of predator presence and habitat composition on occupancy patterns of these species are unknown.

Here, we analyze, at two different spatial scales, the effects of predation risk and habitat composition as well as their interaction on occupancy of nest boxes by near threatened flying squirrel and more common, but also declining red squirrel in central Finland. We hypothesize that predator presence has a negative effect on nest box occupancy by arboreal squirrels, either due to lethal or nonlethal effects, and increasing proportion of preferred habitat within home range has a positive one. Based on the concept of predation risk landscape, we hypothesize that in the presence of a predator, squirrels use less of a mutually preferred habitat. Habitat specific predation could also decrease occupancy of boxes in some habitat in the presence of predators. The habitat use of red squirrels also depends on food abundance, because red squirrels shift their diet from cones of Norway spruce (*Picea abies*) to those of Scots pine (*Pinus sylvestris*) if the former are not available in sufficient amounts [29]. Therefore we hypothesize variations in cone abundance to be reflected on the occupancy patterns of the nest boxes in different forest types. For flying squirrels, we are unable to make similar hypothesis of changes in habitat use in relation to variation in food levels.

Based on above hypotheses, we predict that (i) nest box occupancy by flying and red squirrels is lower where the predation risk index is higher, and occupancy increases with increasing area of a high-quality habitat within the buffers. We also anticipate the strength of these effects to reflect the relative roles of predators and habitat on site occupancy. In addition, we (ii) predict interactive effects between habitat and predation risk on nest box occupancy. For red squirrels, we predict (iii) occupancy rate of nest boxes in spruce forest to be highest and that in pine forests to be relatively lower in years with abundant spruce cone crop, and the opposite (low occupancy in spruce and high in pine forest) to be true in spruce cone crop failure years.

## Material and methods

### Study species

The flying squirrel is a nocturnal sciurid which lives in mature boreal spruce-dominated mixed forests feeding on buds, leaves, catkins, and seeds of deciduous trees [30]. It is listed on the annexes II and IV of the EU Habitats and Species Directive, which implies strict protection of the resting and reproduction sites of the species. The flying squirrel has declined in Finland during the past decades [27, 28, 31] and habitat loss is considered the main reason behind the decline (e.g. [28, 32–34]). It is currently listed as near threatened in Finland [35]. The main predator of the flying squirrel in Eurasian boreal forests is the nocturnal Ural owl (*Strix uralensis*), but also diurnal Northern goshawks (*Accipiter gentilis*, hereafter goshawk) prey on flying squirrels [27]. Because goshawks also prey on and compete with Ural owls [36], their presence can also benefit flying squirrels [37], and thus the effect of goshawk on its prey species is not necessarily a linear one. Both birds of prey prefer mature mixed and spruce-dominated forest [23, 36, 38, 39] as their habitat, just like the flying squirrel.

The diurnal red squirrel is a far more common species than the flying squirrel, and it is wide-spread in temperate and boreal forest ecosystems throughout Eurasia, bur recent studies indicate that it also has been declining [27, 40]. In boreal coniferous forests, the main food of the red squirrel is seeds of Norway spruce [41, 42]. However, red squirrels can adjust their diet to the availability of different food resources [29, 43], which may affect their space use [44]. When spruce seeds are scarce, seeds of Scots pine are an important substitute in boreal forests [29]. Fluctuations in conifer seed abundances cause synchronous fluctuations in squirrel numbers [42, 44, 45]. The main predator of the red squirrel in our study area is the diurnal goshawk [23], and red squirrels make up about 10% of their diet [27].

### Flying squirrel and red squirrel data

**Fig 1:**
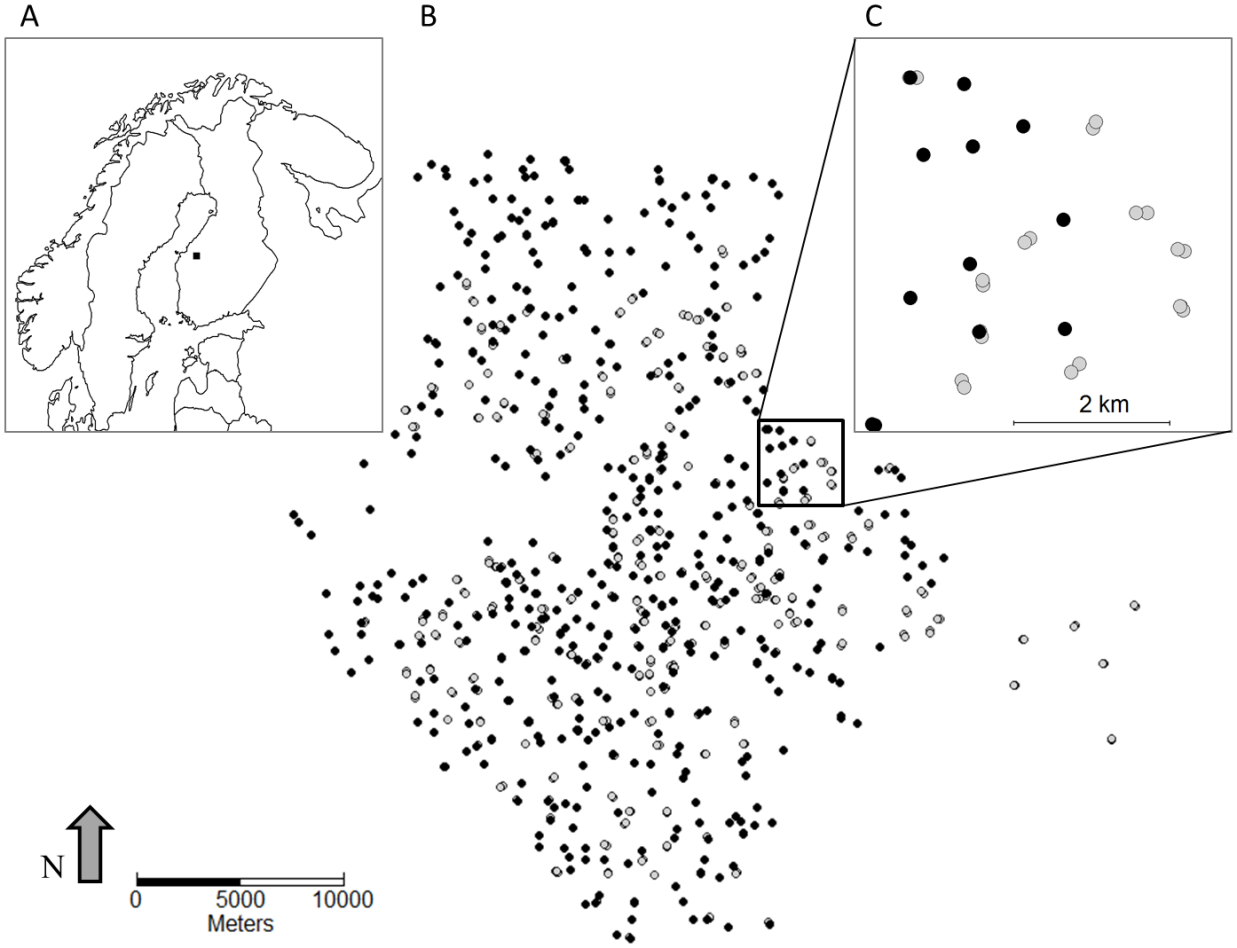
The spatial distribution of red and flying squirrel nest boxes in the study area. (A) Black square shows the location of study area in Finland at approximately 62° 54’– 63° 16’ N, 22° 54’– 23°47’ E. (B) All the boxes censused in the year 2010. Open symbol: flying squirrel nest box, filled symbol: red squirrel nest box. (C) A close up showing how there are two flying squirrel nest boxes (light grey) per site and one red squirrel nest box (black) per site.

The study area is located in the Kauhava region, western Finland (62° 54’– 63° 16’ N, 22° 54’– 23°47’ E; Fig 1A), where the landscape is mainly characterized by a mosaic of commercially managed coniferous forests, agricultural fields, and peatland bogs. Some mixed and old-growth forests as well as many clear-cuts and sapling areas are also found within the area. The area is sparsely populated and settlement mainly consists of one-family houses and farmhouses.

Both squirrel species utilize nest boxes when available. In absence of human influence, red squirrels build dreys, whereas flying squirrels are dependent on natural cavities, which have become scarce in Finnish managed forests, and also in our study area [17, 46]. In the study area, flying squirrels use nest boxes built for Pygmy owls (*Glaucidium passerinum*) (see e.g. [28, 30, 33, 47]). This nest box type resembles cavities made by the great spotted woodpecker (*Dendrocopos major*) with the thickness of front wall >50 mm and the diameter of the entrance-hole of 45 mm. The nest- boxes are made to resemble natural cavities by using trunk of spruce or aspen (*Populus tremula*) instead of board. Nest boxes are grouped so that there are 2 boxes (80–100 m apart) within a forest site, the sites being at least 0.8–1.0 km apart (Fig 1B and 1C). The boxes are located in various forest types and are moved to new site if their immediate surrounding is clear-cut. The occupancy of the boxes by flying squirrels was checked at least once every spring in 2002–2015. In cases with multiple visits to nest box, we used only the result (presence/absence) of the first visit to reach equal search effort for all nest boxes, and only included nest boxes that had been checked by the end of June. Detection probability in different forest types does not differ substantially, because natural cavities are scarce and are likely to have very little effect on the use of nest boxes, which are far more abundant in studied forest area [17, 46].

Red squirrels were similarly monitored during 1999–2015 by checking for the presence of the animals themselves or their nest material in nest boxes between March and June. The nest boxes are designed for Tengmalm’s owls (*Aegolius funereus*), but are of suitable size and are often used by red squirrels [42, 48]. Furthermore, occupancy of nest boxes by red squirrels correlates positively with snow track index of their abundance, suggesting that occupancy rate reflects true changes in population size [42]. Red squirrel nest boxes are distributed in the landscape at an average density of 0.5 boxes per km^2^. The monitoring of nest boxes for the presence of red squirrels became more rigorous as from 2006 (since this year also presence of nest material in nest boxes was reported; [42]), and therefore the squirrel data have the variable ‘time period’ describing the differing detection probabilities before and since 2006. During this study, on average 357 Pygmy owl boxes (in total 578 different boxes during study years) and 415 Tengmalm’s owl boxes (563 different boxes) were checked every year.

### Habitat data

The areas of different land use classes (Table 1) within buffers of 200 and 1000 meters (for flying squirrel) and 300 and 2500 meters (for red squirrel) were calculated for each nest box in ArcMap 10.1 and R 3.2.5 [49]. The smaller buffers correspond roughly to estimated female home-range size [50–52] and larger buffers describe landscape at scale of dispersal movements for both species within our study area [53]. Thus, the selected spatial scales capture habitat composition at the levels central for squirrel reproductive success (the smaller scale) and movement ability (the larger scale). Landscape maps were based on SLICE dataset [54], two forest classifications, from 1997 (National Land Survey, [55]) and 2009 [56], and Landsat Images (http://landsat.usgs.gov/), so that yearly changes in forest cover (e.g. clear-cutting of forest) were taken into account. For a detailed description of map processing, see [17].

**Table 1:** Land use classes, their combinations and abbreviations.

Code	Description	Combinations	Abbreviation
1	Clear cuts		Clear cut
3	Young birch dominated	Young birch	Y birch
6	Young birch pine		
4	Young pine dominated		Y pine
5	Young spruce dominated	Young spruce/mixed	Y mix
7	Young pine spruce		
8	Young birch spruce		
9	Mature birch dominated	Mature birch	Mo birch
15	Old birch dominated		
10	Mature pine dominated	Mature pine	Mo pine
16	Old pine dominated		
11	Mature spruce dominated	Mature spruce	Mo spruce
17	Old spruce dominated		
12	Mature birch pine	Mature birch pine	Mo birch pine
18	Old birch pine		
13	Mature pine spruce	Mature pine spruce	Mo pine spruce
19	Old pine spruce		
14	Mature birch spruce	Mature birch spruce	Mo birch spruce
20	Old birch spruce		
21	Built up	Built environment	Built
22	Roads		
23	Mines		
24	Peat bogs and similar		Bog

The names of land use classes listed in the 4^th^ column (Abbreviation) were used in models for flying and red squirrel data.

### Estimating the impact of predatory birds

Data on Ural owls and goshawks were collected by surveys on natural cavities, nest boxes and large stick nests and by searching for new nest sites annually. Long-term studies of birds of prey are carried out in the Kauhava region (e.g. [17, 48]), so the locations of raptor nests are well known. Density of Ural owls was approximately 2 pairs per 10 km^2^, and that of goshawks 1 pair per 10 km^2^ in the study area [48]. Due to lack of data on Ural owls from the year 2001, same values as in 2002 were used. The usage of same values for two years is justifiable because Ural owls’ longevity and site-fidelity result in territories remaining occupied for a long time [57]. Predation risk was assumed to be highest close to the raptor nest and to remain at a high level within a given distance and to then decrease symmetrically to all directions when moving further from the nest (see next paragraph for details). Both Byholm et al. [37] and Selonen et al. [58] have shown flying and red squirrels, respectively, to be negatively affected by the location of the nests of their main avian predators. This supports our assumption that predation risk from avian predators for arboreal squirrels is related to location of nests of the predators. *Accipiter* hawks have been found to exhibit a random pattern of returning to the same hunting spot, maximizing the unpredictability of attacks by their prey [59, 60], which supports the use of symmetrically decreasing predation risk to all directions.

Goshawk (for both squirrel species) and Ural owl (for flying squirrel) presence at squirrel observation points (nest box sites) was modelled by calculating flat-top bivariate Gaussian kernels around nests of birds of prey, using the same method that Björklund et al. [61] successfully used to model goshawk threat for intraguild prey. The flat-top part represents the area where the impact of the avian predators is strongest, beyond which it declines following the Gaussian distribution until a cut-off distance of 10 km. The height of the kernel at flying or red squirrel nest box location was used as a proxy for predation risk. Kernels with *SD* of 1,2,3, and 4 and flat tops of 500, 1000, 1500, 2000, and 2500 m and all their combinations were compared and the one that explained the presence/absence of squirrels most parsimoniously (as measured with model AIC) was used. The kernel explaining most flying squirrel presence for goshawk was a kernel with *SD* of 3 and flat top of 2500 m and for Ural owl with *SD* of 4 and flat top of 500 m. A goshawk kernel with *SD* of 1 and flat-top of 500 m explained red squirrel occupancy best. Because the effect of predators on squirrel nest site occupancy may be lagged (predatory effect from previous year) or immediate (e.g. antipredator behavior), we calculated kernel values using Ural owl and goshawk data both from current and previous year. Based on AIC comparison of full models, previous year’s Ural owl and goshawk kernels were selected for final models (difference in AIC 1.7 (2663.0–2661.3)) for flying squirrel. For red squirrel, current year’s goshawk kernel was selected (difference in AIC 0.8 (6710.9–6710.1)) instead.

### The effect of spruce cone crop on habitat use of red squirrels

For spruce cone crop, we used data provided by the Natural Resources Institute Finland (Luke). Cones are counted yearly in research forests thorough Finland [62]. We used estimates of yearly cone crop from 3 nearest research forests within ca. 75 km from the mean coordinates of the study area. In other words, we did not have data for cone crop within the study area, but spruce cone crop is strongly spatially autocorrelated at distances of a few hundred kilometers (e.g. [63]), and the used estimate describes well the yearly fluctuations in cone crop in our study area [42]. We used the cone crop estimate of the previous year, counted in late autumn, in relation to red squirrel data, because this cone crop affects the red squirrel numbers in the following winter and spring (e.g. [41, 42]), i.e. at the time when the nests of red squirrels were built to the nest boxes monitored in the current study.

### Analyses

We built 2 main models per species to analyze the occupancy by the two squirrel species at the two spatial scales (small and large). All data management and analyses were done in R.

We used binomial generalized linear mixed models with nest box ID and site ID as nested random factors (two nest boxes per site in flying squirrel data) or only site ID as a single random factor (1 nest box per site in red squirrel data). Models were run using the function ‘glmer’ in the package ‘lme4’ [64]. All continuous explanatory variables, including year, were standardized as ((x-mean(x))/sd(x)) [65]. The quadratic effect of field was included in the models, because previous studies suggest a positive association between farmland and flying squirrel occurrence [66], but obviously there has to be an upper limit for the proportion of farmland in the landscape for forest animals. Multicollinearity in the data was assessed with variance inflation factors (VIFs, calculated using the package ‘car’ [67]), and young pine forest was discarded from both flying and red squirrel models due to large VIF. After removing it, VIFs of all remaining variables were below 5 in flying and red squirrel models, which was considered an acceptable level. Values between 5 and 10 are often used [68, 69], but recommendations vary considerably. The effect of young pine forest on flying and red squirrel occupancy within both small and large buffers was studied with separate models. We also compared the results of the full model to results of 10 best models based on model selection with the function ‘glmulti’ (package ‘glmulti’; [70]). Because not all of the variables that were a priori of interest were included in the best models, we used the full model in the main analysis.

To study the possible interactive effects of predators and habitat, interaction terms between predator kernel value and preferred and the most non-preferred habitat were added to small (home range) scale models. We used the habitat which had the strongest negative impact as non-preferred habitat, and as preferred habitat we used mature forests for flying squirrels and mature spruce forests for red squirrels. Non-significant interactions were dropped from final models. To study the effect of food availability on habitat use of red squirrels, we included the interaction between yearly cone crop estimate of the previous year and amount of spruce or pine dominated forests to separate models where site ID was a random factor. In other words, there were 2 models for this purpose: a model with cones*pine forest interaction and a model with cones* spruce forest interaction, to test the prediction iii) that red squirrels shift from spruce- to pine-dominated forests when spruce cone abundance is low.

We measured spatial autocorrelation with Moran’s I of the model residuals (package ‘ape’ [71]) from radii 50, 500 and 5000 m from flying and red squirrel nest boxes, and at all radii the autocorrelation was negative and very small, reaching the smallest value I = -0.03 at the distance of 500 m. The values were similar for all flying and red squirrel model residuals. Thus, we did not need to account for spatial autocorrelation in the current analysis.

## Results

### Nest box occupancy of flying squirrel

**Fig 2:**
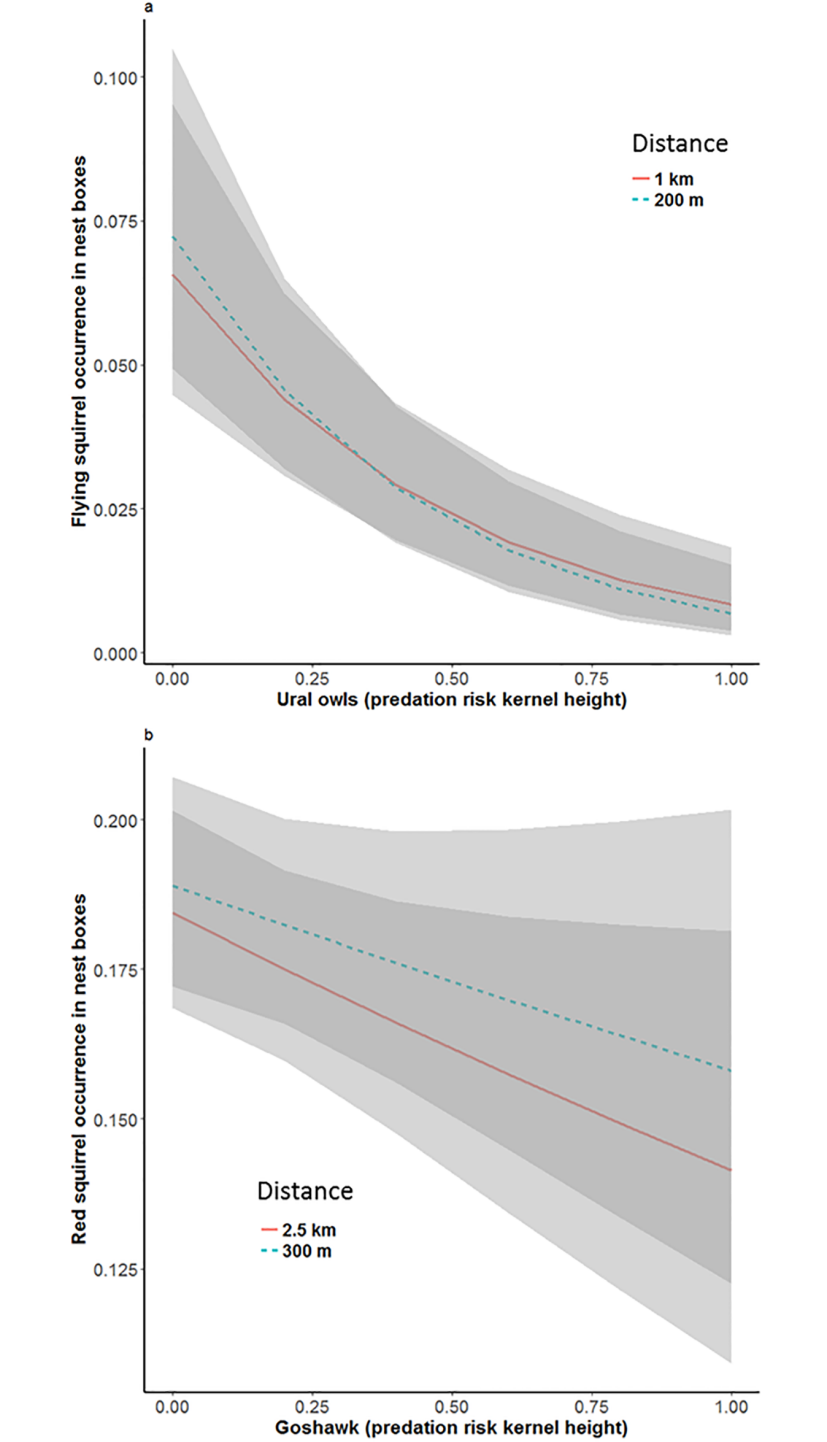
The presence of main avian predator decreases nest-box occupancy by flying and red squirrels. The effect of (A) Ural owl (kernel height) on occurrence of flying squirrels and (B) goshawk (kernel height) on occurrence of red squirrels in nest boxes based on models at small and large spatial scale.

**Fig 3:**
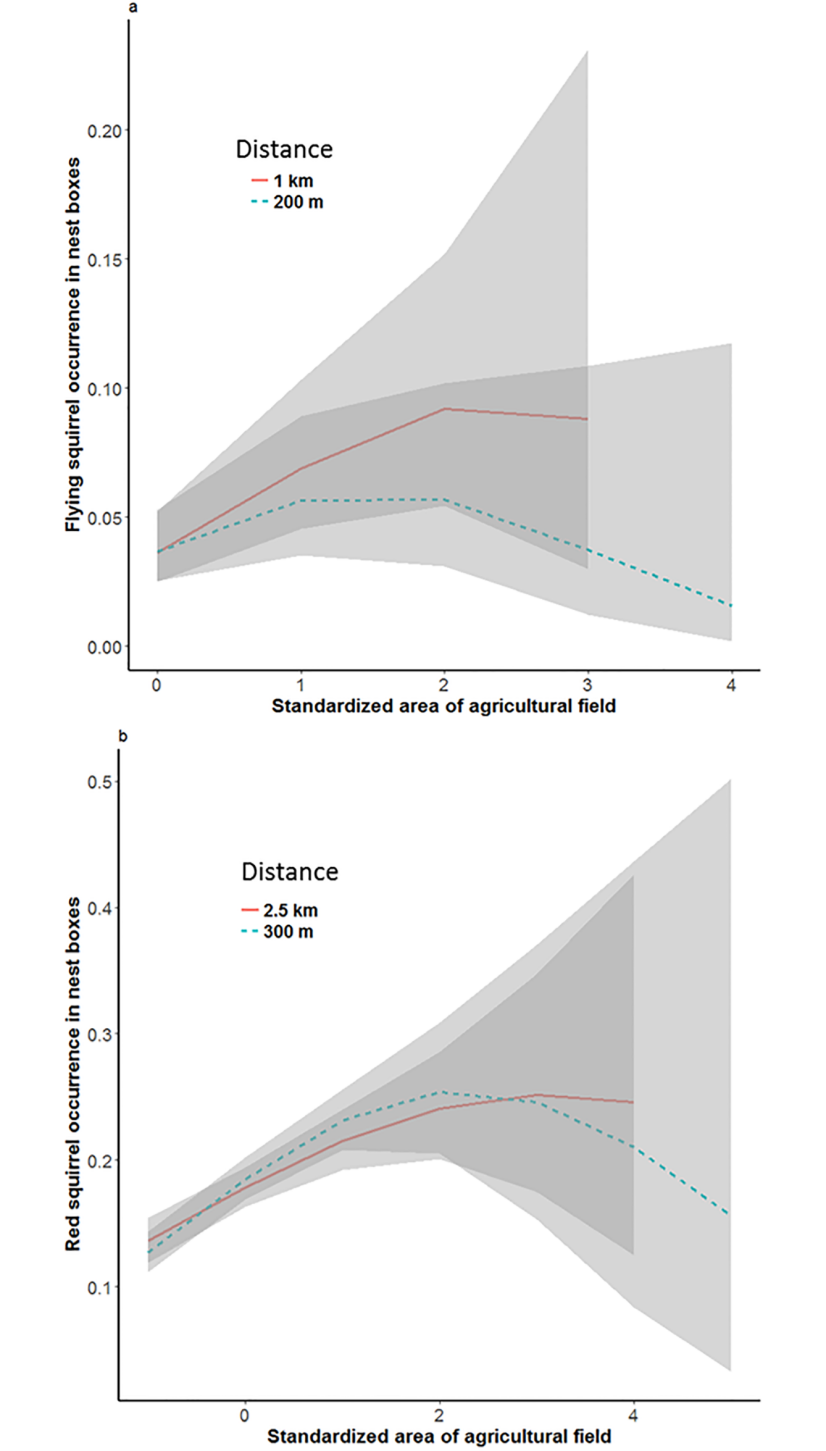
Increasing area of field first increases and eventually decreases occurrence of squirrels. The effect of standardized amount of agricultural field (A) within buffers of 200 m and 1 km on flying squirrel occurrence in nest boxes, and (B) within buffers of 300 m and 2.5 km on red squirrel occurrence in nest boxes.

As predicted, Ural owl risk had a strong negative impact on flying squirrel occurrence in nest boxes (Fig 2A, Table 2). Surprisingly, the expected positive associations with preferred habitat types were not detected (Table 2). Instead, farmland had the expected positive effect at both small and large spatial scale (200 and 1000 m, respectively). The quadratic term for farmland was significantly negative at both scales, suggesting that there is an optimum for the amount of farmland, small and large values correlating with smaller occupancy probability of nest boxes (Fig 3A). The mean areas of different land use classes around nest boxes are shown in S1 Table. Flying squirrel occurrence increased with time (Table 2, S2 Table) from 8 occupied nest boxes (8% occupied) in 2002 to 53 occupied boxes (13%) in 2015. The predicted non-linear relationship between flying squirrel and goshawk was not supported (Table 2). The alternative models based on model selection provided additional support for the effects of Ural owl and farmland on flying squirrels, as these were included in the best models at both scales (see S3 Table for the output of model selection).

**Table 2:** Summary of flying squirrel models.

Model	Covariate	Estimate (log odds) ± *SE*	Test
F1	Intercept	-0.09±1.13	*z* = -0.08, *P* = 0.94
	**Year**	**0.26±0.06**	***z* = 4.22, *P* < 0.001**
	Clear cut	-0.03±0.12	*z* = -0.26, *P* = 0.8
	Y birch	0.08±0.13	*z* = 0.59, *P* = 0.56
	Y mixed	0.13±0.1	*z* = 1.29, *P* = 0.2
	Mo birch	-0.11±0.13	*z* = -0.9, *P* = 0.37
	Mo pine	-0.03±0.14	*z* = -0.24, *P* = 0.81
	Mo spruce	0.05±0.12	*z* = 0.42, *P* = 0.68
	Mo birch pine	0.18±0.12	*z* = 1.49, *P* = 0.14
	Mo pine spruce	0.04±0.12	*z* = 0.3, *P* = 0.76
	Mo birch spruce	-0.11±0.13	*z* = -0.86, *P* = 0.39
	Built	-0.11±0.11	*z* = -0.97, *P* = 0.33
	Bog	0.001±0.14	*z* = -0.01, *P* = 0.99
	**Ural owl** **(at year**_**t-1**_**)**	**-2.41±0.45**	***z* = -5.31, *P* < 0.0001**
	**Field**	**0.71±0.19**	***z* = 3.64, *P* < 0.001**
	**Field**^**2**^	**-0.23±0.1**	***z* = -2.42, *P* = 0.02**
	Goshawk (at year_t-1_)	-3.48±2.8	*z* = -1.24, *P* = 0.21
	Goshawk^2^ (at year_t-1_)	0.87±1.87	*z* = 0.47, *P* = 0.64
F2	Intercept	0.03±1.05	*z* = 0.03, *P* = 0.97
	**Year**	**0.24±0.07**	***z* = 3.33, *P* < 0.001**
	Clear cut	0.09±0.15	*z* = 0.59, *P* = 0.55
	Y birch	-0.23±0.23	*z* = -1, *P* = 0.32
	Y mixed	-0.23±0.18	*z* = -1.29, *P* = 0.2
	Mo birch	0.003±0.17	*z* = -0.02, *P* = 0.98
	Mo pine	-0.12±0.18	*z* = -0.64, *P* = 0.52
	Mo spruce	0.05±0.12	*z* = 0.43, *P* = 0.67
	Mo birch pine	0.06±0.21	*z* = 0.27, *P* = 0.79
	Mo pine spruce	-0.09±0.16	*z* = -0.54, *P* = 0.59
	Mo birch spruce	0.28±0.16	*z* = 1.75, *P* = 0.08
	Built	-0.24±0.13	*z* = -1.84, *P* = 0.07
	Bog	-0.14±0.13	*z* = -1.06, *P* = 0.29
	**Ural owl** **(at year**_**t-1**_**)**	**-2.1±0.44**	***z* = -4.74, *P* < 0.0001**
	**Field**	**0.89±0.17**	***z* = 5.3, *P* < 0.0001**
	**Field**^**2**^	**-0.19±0.1**	***z* = -2.01, *P* = 0.04**
	Goshawk (at year_t-1_)	-4.4±2.66	*z* = -1.66, *P* = 0.1
	Goshawk^2^ (at year_t-1_)	1.59±1.81	*z* = 0.88, *P* = 0.38

In model F1 habitats were measured within 200 meters and in F2 within 1000 meters from nest boxes. Effects in bold indicate a significant P-value (P < 0.05). Letters in covariate names refer to forest age: Y = young, Mo = mature and old. The supercripts ^2^ refer to quadratic terms.

**Fig 4:**
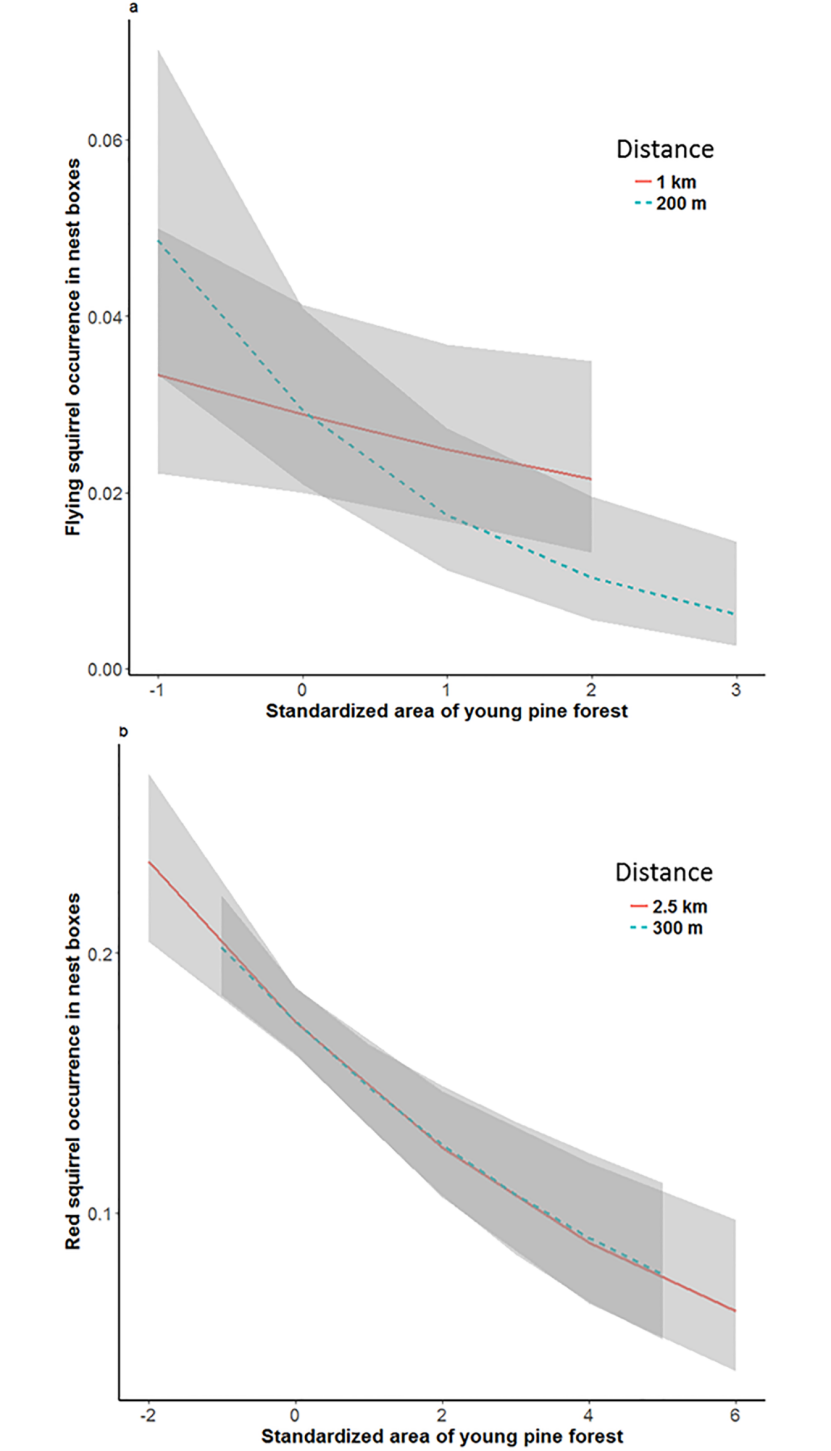
Young pine forest has a negative effect on nest box occupancy by arboreal squirrels. The effect of standardized amount of young pine forest (A) within buffers of 200 m and 1 km on flying squirrel occurrence in nest boxes, and (B) within buffers of 300 m and 2.5 km on red squirrel occurrence in nest boxes.

The effect of young pine forest on flying squirrel occurrence, analyzed in separate models to avoid multicollinearity, was negative (Fig 4A) on small (estimate -0.37±0.15, *z* = -2.5, *P* = 0.01) but not at large scale (estimate -0.15±0.09, *z* = -1.68, *P* = 0.09).

### Nest box occupancy of red squirrel

**Fig 5:**
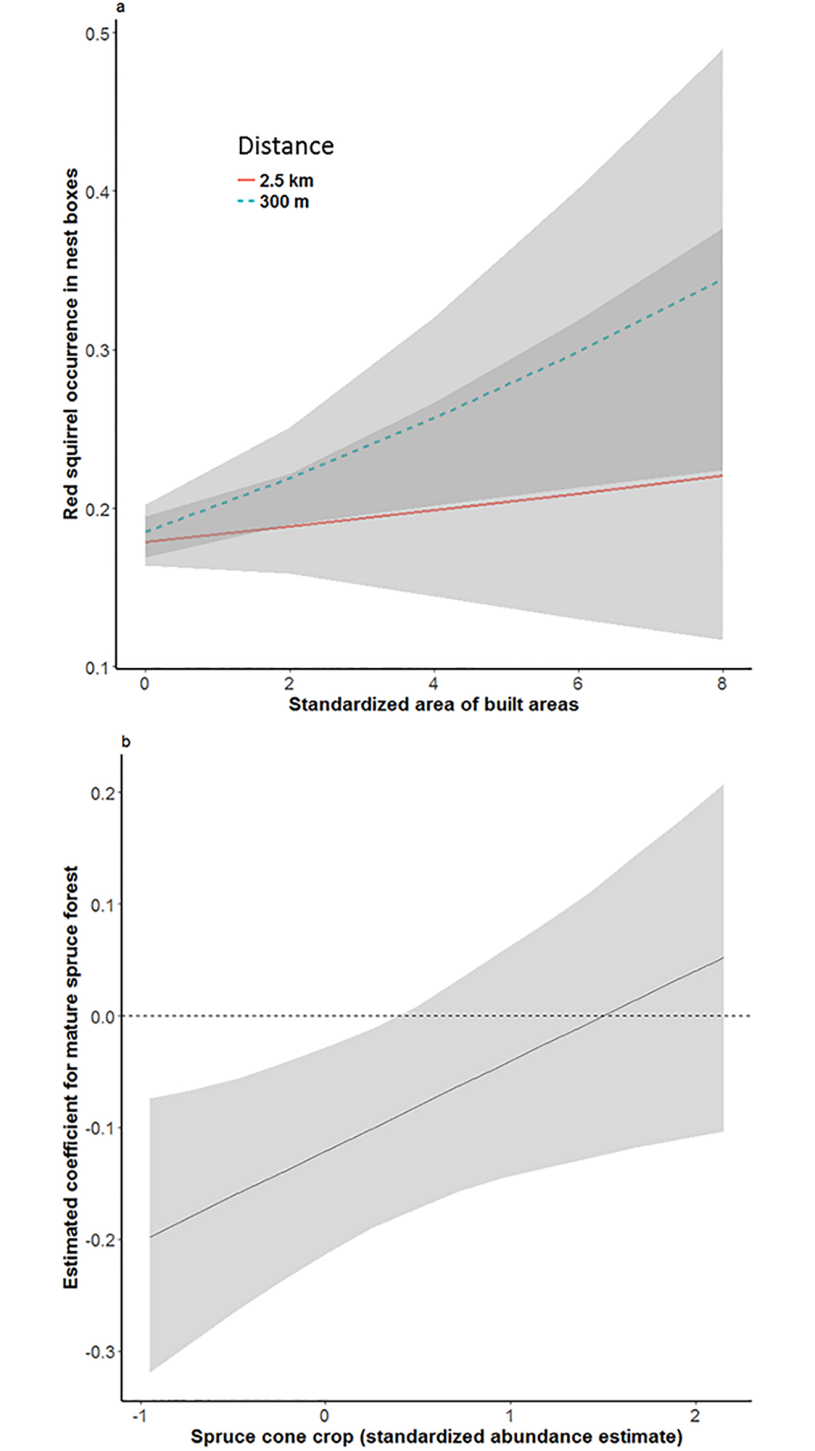
Red squirrels benefit from human settlement, and nest boxes in mature and old spruce forests are more likely to be occupied when spruce cone crop is abundant. The effect of (A) standardized amount of built areas within buffers of 300 and 2.5 km on red squirrel occurrence in nest boxes and (B) the effect of spruce cone crop on the estimated coefficient of mature spruce forest, i.e. the interaction between cone crop and spruce forest on red squirrel occurrence.

The effect of goshawk predation risk on red squirrel occurrence was negative and significant at large scale (Fig 2B; Table 3). Similarly as for flying squirrels, the expected association with preferred habitat was not detected (Table 3). Instead, red squirrel occurrence was positively associated with amount farmland at both small (300 m) and large (2500 m) scale, and also with built-up areas at the small scale (Figs 3B and 5A; Table 3). The quadratic term of farmland was negative and significant at small scale. At large scale, mature birch-spruce forest had a negative impact on red squirrel occurrence. Nest box occupancy of red squirrels decreased with time (S2 Table). Young pine forest, which was not included in the main model, had a negative effect at both small (estimate -0.14±0.05, *z* = -3.17, *P* < 0.01) and large (estimate -0.19±0.04, *z* = -4.74, *P* < 0.0001) scale (Fig 3B). Output of the model selection (S3 Table) further confirmed the positive effects of built areas at small scale and farmland at both scales, as these are included in the best models within 2 AIC units.

**Table 3:** Summary of red squirrel models.

Model	Covariate	Estimate (log odds) ± *SE*	Test
R1	Intercept	-2.27±0.1	*z* = -22.37, *P* < 0.0001
	**Year**	**-0.16±0.06**	***z* = -2.65, *P* = 0.01**
	**Time period 2**	**1.33±0.12**	***z* = 10.71, *P* < 0.0001**
	Clear cut	0.02±0.05	*z* = 0.44, *P* = 0.66
	Y birch	-0.01±0.05	*z* = -0.15, *P* = 0.88
	Y mixed	0.06±0.04	*z* = 1.3, *P* = 0.19
	Mo birch	-0.03±0.05	*z* = -0.51, *P* = 0.61
	Mo pine	0.05±0.05	*z* = 0.93, *P* = 0.35
	Mo spruce	-0.05±0.05	*z* = -1.06, *P* = 0.29
	Mo birch pine	-0.06±0.05	*z* = -1.09, *P* = 0.28
	Mo pine spruce	-0.04±0.05	*z* = -0.73, *P* = 0.47
	Mo birch spruce	-0.06±0.05	*z* = -1.11, *P* = 0.27
	**Built**	**0.11±0.04**	***z* = 2.75, *P* = 0.01**
	Bog	-0.1±0.05	*z* = -1.95, *P* = 0.05
	**Field**	**0.36±0.06**	***z* = 6.5, *P* < 0.0001**
	**Field**^**2**^	**-0.08±0.04**	***z* = -2.16, *P* = 0.03**
	Goshawk (at year_t_)	-0.22±0.16	*z* = -1.39, *P* = 0.16
R2	Intercept	-2.28±0.1	*z* = -22.72, *P* < 0.0001
	**Year**	**-0.17±0.07**	***z* = -2.46, *P* = 0.01**
	**Time period 2**	**1.3±0.12**	***z* = 10.42, *P* < 0.0001**
	Clear cut	0.04±0.07	*z* = 0.54, *P* = 0.59
	Y birch	0.15±0.09	*z* = 1.63, *P* = 0.1
	Y mixed	0.14±0.09	*z* = 1.61, *P* = 0.11
	Mo birch	-0.15±0.08	*z* = -1.8, *P* = 0.07
	Mo pine	-0.13±0.07	*z* = -1.78, *P* = 0.07
	Mo spruce	0.002±0.06	*z* = -0.03, *P* = 0.98
	Mo birch pine	0.05±0.09	*z* = 0.59, *P* = 0.55
	Mo pine spruce	-0.07±0.07	*z* = -0.92, *P* = 0.36
	**Mo birch spruce**	**-0.21±0.06**	***z* = -3.32, *P* < 0.001**
	Built	0.03±0.05	*z* = 0.67, *P* = 0.5
	Bog	0.02±0.06	*z* = 0.35, *P* = 0.73
	**Field**	**0.28±0.06**	***z* = 4.63, *P* < 0.0001**
	Field^2^	-0.04±0.03	*z* = -1.39, *P* = 0.17
	**Goshawk** **(at year**_**t**_**)**	**-0.32±0.16**	***z* = -2.02, *P* = 0.04**

In model R1 habitats were measured within 300 meters and in R2 within 2500 meters from nest boxes. Effects in bold indicate a significant P-value (P < 0.05). Letters in covariate names refer to forest age: Y = young, Mo = mature and old. The superscripts ^2^ refer to quadratic terms.

### Interactive effects between habitat, predators, and food

We did not detect significant interaction between area of preferred habitat and presence of predators on flying squirrel (Ural owl*mature forests: estimate -0.09±0.35, *z* = -0.26, *P* = 0.79) or red squirrel (goshawk*mature spruce forest: estimate -0.14±0.14, *z* = -0.99, *P* = 0.32) occurrence. The interaction term between the significantly non-preferred habitat, young pine forest, and the main predator, the Ural owl, did not affect occurrence of flying squirrels (estimate -0.53±0.41, *z* = -1.28, *P* = 0.2), but for red squirrels, interaction between young pine forest and goshawk was significant (estimate -0.36±0.16, *z* = -2.17, *P* = 0.03).

The interaction between yearly cone crop estimate and area of spruce forest (estimate 0.08±0.04, *z* = 2.30, *P* = 0.02, Fig 5B) had a slight positive impact on red squirrel occurrence, whereas interaction between cone crop and pine forest did not (estimate 0.005±0.03, *z* = 0.17, P = 0.87). The positive effect of cone crop per se was strong (estimate 0.17±0.03, *z* = 5.41, *P* < 0.0001).

## Discussion

Our results suggest that nest-box occupancy of night-active flying squirrels and day-active red squirrels was clearly negatively affected by the presence of avian predators that move and hunt when the squirrels are active. The predicted positive associations with preferred habitat types were not detected, while young pine forest seemed to be an avoided habitat for both squirrel species. Both squirrel species were positively associated with close proximity to farmland up to a certain threshold. In contrast to our expectation, there was no obvious indication of trade-off between predation pressure and settling in a high-quality habitat. However, the effect of goshawk presence on red squirrels increased to become significantly negative if there was much young pine forest around nest box, in line with the predicted habitat specific predation. Lastly, red squirrels seemed to adapt their nest site use based on fluctuations of spruce cone crop.

The result that Ural owls had a significant negative effect on flying squirrels is consistent with the North American studies concluding that certain owl species may have clear negative impact on local flying squirrel species. Northern flying squirrels (*Glaucomys sabrinus*) are the main prey of spotted owls (*Strix occidentalis*), and their predation pressure is strong enough to decrease Northern flying squirrel populations [22, 72]. Whether or not the observed effect of Ural owls on Siberian flying squirrel nest occupancy translates to decrease in population size requires further studies. The yearly predator numbers are not significantly related to flying squirrel nest occupancy rates in our data. However, the data is not optimal for assessing this effect, because Ural owls are resident on their territories also in poor vole years [73], limiting variation in owl numbers. In any case, the clear negative impact of Ural owls on flying squirrels raises the question whether erecting nest box networks for Ural owls should be avoided in the vicinity of known or potential flying squirrel territories. Ural owls are known to reduce the reproductive success of Tengmalm’s owls and predate them up to 2 km from Ural owl nests [48, 74], and may threaten flying squirrels in a similar manner. Ural owls are classified as least concern while flying squirrels are near threatened [35], so it seems reasonable to prioritize the conservation of flying squirrels and to locate nest boxes for the owls elsewhere.

The predation risk landscape did not affect the type of habitat individuals used in our study (expect for increased avoidance of young pine forest by red squirrels), that is, squirrels did not seem to be chased away from good-quality habitats, as was predicted. We are unaware of earlier studies on relative roles of habitat and predation risk on site occupancy of arboreal squirrels. Animals usually avoid predators by selecting areas that minimize predation risk at a large scale, and at smaller scale choose habitat based on (also) other limiting factors (e.g. [75, 76]). This hierarchical habitat selection may explain why predators simultaneously had a significant effect and yet no interaction between predator presence and settling in a high-quality habitat was found. It is important to remember that in both our small and large scale models the index value for predators was extracted for nest box location, and hence did not vary between the models. Previous studies have found predators or predation risk landscapes to affect habitat selection of various types of prey species, for example browsers [77], passerine birds [3], hedgehogs [78], and deer [79]. Studies that have compared the relative effects of predation and habitat type on mammal space use have also highlighted the crucial effect of predator avoidance [12, 18, 19, 80]. In contrast to these studies, the trade-off between predator avoidance and settling in a high-quality habitat seems less noticeable in nest box use of arboreal squirrels, perhaps because nest boxes cannot be entered by Ural owls and goshawks and thus offer refuges against the large avian predators.

The negative effect of goshawk presence on red squirrel occurrence was less clear than the effect of Ural owl on flying squirrels. However, in the earlier open population experiment performed in the same study area, Selonen et al. [58] concluded that avian predators have a clear effect on red squirrels. They found that during winter, when natural food was scarce, abundance indices of red squirrels decreased at sites with predators. Previous observational studies in more southern areas have concluded that while goshawks prey on red squirrels regularly, they apparently do not limit red squirrel numbers [45, 81]. It may be that the effect of predators on red squirrels is determined by not only predator abundance, but also habitat type and quality and season, which would explain the slightly differing results of studies conducted in different areas.

Naturally, the real predation risk perceived by animals is formed by all the predators in the area. The avian predators studied here are not the only predators of the squirrels in the study area, but they are considered the most important ones. For example, the pine marten (*Martes martes*) preys on red squirrels, but it’s density is very low in the Kauhava study area based on snow track records [58] and the low proportion of bird nests predated by pine martens (<5%, [48]). Cats (*Felis catus*) and red foxes (*Vulpes vulpes*) that hunt on the ground can catch squirrels practically only in open areas (e.g. in fields and built areas) where squirrels move more on the ground, and are not important predators in forests.

Unexpectedly, the area of spruce forest did not increase the probability of nest box occupancy by red or flying squirrels. It seems that both squirrels can actually be quite flexible in their habitat use. This is in contrast to studies that have found flying and red squirrels to primarily prefer mature and old spruce dominated forests (e.g. [66, 82, 83]). On the other hand, there are also studies that conclude flying and red squirrels to be quite flexible in their habitat use [84, 85]. Important difference between our study and most of the earlier ones is that in our case nest sites, i.e. nest boxes, were not a limiting resource, and that Ural owls and goshawks cannot enter these nests. Similar to our observations, Wheatley et al. [86] found that Northern flying squirrels are rather habitat generalists than specialists, because no forest landscape variable explained their abundance (see also [87]). Wheatley et al. [86] argued that Northern flying squirrels in their study area in Alberta, Canada may respond to stand-level microclimate and food availability, and therefore no association was found between abundance and forest type (but see e.g. [88] for significant effects of forest type on Northern flying squirrels). It remains possible that also in our study population flying squirrels responded to some characteristic of their environment that is not included in forest composition and age. For example, mast of alder (*Alnus sp*.) and birch (*Betula* sp.), primary food resources of flying squirrels in winter, are an important determinant of reproductive success in flying squirrels [85, 89]. Our results also indicate that flying squirrels may be less selective in their habitat use in areas where availability of nest sites is not a limiting factor.

Open farmland had a positive effect on both study species, supporting the earlier observations from flying squirrels [66]. We expected there to be an upper limit for the amount of open habitat, and indeed, when the proportion of agricultural fields exceeded roughly 50% of the landscape on either scale, farmland turned non-beneficial for flying squirrels. Apparently at that point landscape becomes too fragmented or the amount of forest (and forest-related resources) is simply too low. We agree with Santangeli et al. [66] that the apparent positive association with farmland may result from the fact that agricultural fields are usually established on rich soil, and that forest edges are often dominated by deciduous trees that produce food for flying squirrels. We found similar pattern also for red squirrels, which seemed to tolerate or benefit from even larger proportion of agricultural fields than flying squirrels, up to 70% of landscape within home range scale (300 m buffer).

Surprisingly, built areas slightly increased the probability of red squirrel occurrence in our rural study area. Red squirrels are known to adapt well to urban areas and to utilize resources provided by humans [90]. Feeding birds is popular in the Finnish countryside, and red squirrels commonly visit the feeding sites. In general, previous studies on local effects of supplementary feeding on red squirrels and other squirrel species have yielded mostly positive results [84, 91, 92, but see 58]. Built area had no significant effect in the full model for flying squirrels, but it was included in the best models based on model selection and was significantly negative in those (S3 Table). Interestingly, the relationship was opposite to that of red squirrel models, possibly indicating differences in response to built or urban areas between these two arboreal squirrel species.

For red squirrels, we found support, albeit not very strong, for the effect of food availability on nest site use: individuals used nest boxes with more spruce forest around them when spruce cone crop was abundant, but were not found more often in pine forests when spruce cone crop was low. We interpret this so that red squirrels did not shift their nest sites between pine and spruce forests, but dwelt more in spruce forests when they provided ample food supply, as was expected. In addition, it is likely that individuals utilized spruce and pine trees within their home range based on which provided more food. Previous studies have found red squirrels to change habitat use within home range [93, 94] and even shift home ranges [93] in response to differences in habitat quality.

Overall, the effects of different landscape variables on nest box occupancy were surprisingly similar for both study species: farmland had a positive effect while young pine forests had a negative effect, and expected preferences towards mature and old forests were not detected. It has to be noted that old forests are rare in the study area, accounting for approximately 7% of total land area within the buffers around nest boxes, and less of the landscape in general. However, our nest boxes are always located in coniferous forests, although of varying area and species composition, and the lack of preference for mature forest in the current study does not mean that the studied squirrel species do not prefer them in general. More likely the results reflect the adaptability of the squirrels: the forests where the nest boxes are located are mostly “good enough” and that combined with the low availability of old forests lead preferences to not be clear enough to be detected with the current method. It is clear that the current forest management practice of leaving a 10–15 m buffer around nests to protect flying squirrels is not sufficient to maintain flying squirrel occupancy [95, 96]. However, the currently insufficient management practices might be improved quite easily, as our results suggest that flying squirrels do not necessarily require large forest patches. The determination of absolute values for this calls for further studies in different densities and forest types. Based on our results, mixed forest structure may also benefit the flying squirrel, like observed for other taxa [97].

Our results are novel in the sense that they show that predators can have a more marked effect on nest site occupancy of flying and red squirrels than the amount of preferred habitat. The results also demonstrate the fundamental negative effect of Ural owls on Siberian flying squirrel occurrence and weaker, albeit significant, negative impact of goshawks on red squirrel occurrence. Erecting nest boxes for Ural owls at distance of <2 km from permanent flying squirrel territories should be avoided in order to conserve these near threatened squirrels. The prediction that predation risk landscape affects use of high-quality habitat was not supported, although weak support for habitat specific predation was observed for red squirrels in a non-preferred habitat. The results of this study also suggest that flying squirrels do not necessarily need old, continuous forests, if nest sites are present in all types of forests. Thus, our study supports the view (e.g. [86]) that successful species management should focus on identifying key resource, food and nest sites, in the landscape. Management focusing only on forest age or type, commonly used proxies in management of forest animals, may in some cases miss these key resources. Importantly, our study shows that managers should also consider the occurrence of enemies (in this case large avian predators) when planning conservation programs for arboreal squirrels.

## Supporting information

S1 Table Areas of different habitat types within small and large buffers around occupied and unoccupied squirrels nest boxes. (DOCX)

S2 Table Yearly occupancy rates of flying and red squirrel nest boxes. (DOCX)

S3 Table Alternative models. (DOCX)

## References

[1] Lima SL. Putting predators back into behavioral predator–prey interactions. Trends Ecol Evol. 2002; 17: 70–75.

[2] Norrdahl K, Korpimäki E. Mortality factors in a cyclic vole population. Proc R Soc Lond B Biol Sci. 1995; 261: 49–53. 10.1098/rspb.1995.0116 7644548

[3] Thomson RL, Forsman JT, Sarda-Palomera F, Mönkkönen M. Fear factor: prey habitat selection in a predation risk landscape. Ecography. 2006; 29: 507–514.

[4] Brown JS, Laundré JW, Gurung M. The ecology of fear: optimal foraging, game theory, and trophic interactions. J Mammal. 1999; 80: 385–399.

[5] Laundré JW, Hernández L, Ripple WP. The Landscape of Fear: Ecological Implications of Being Afraid. Open J Ecol. 2010; 3: 1–7.

[6] Zanette LY, White AF, Allen MC, Clinchy M. Perceived predation risk reduces the Number of Offspring Songbirds Produce per Year. Science. 2011; 334: 1398–1401. doi: 10.1126/science.1210908 22158817 10.1126/science.1210908

[7] Laundré JW, Hernández L, Medina PM, Campanella A, Lopez-Portillo J, Gonzalez-Romero A, et al The landscape of fear: the missing link to understand top-down and bottom-up controls of prey abundance? Ecology. 2014; 95: 1141–1152. 25000746 10.1890/13-1083.1

[8] Lima SL, Dill LM. Behavioral decisions made under the risk of predation: a review and prospectus. Can J Zool. 1990; 68: 619–640.

[9] Schneider MF. Habitat loss, fragmentation and predator impact: spatial implications for prey conservation. J Appl Ecol. 2001; 38: 720–735.

[10] Ryall KL, Fahrig L. Response of predators to loss and fragmentation of prey habitat: a review of theory. Ecology. 2006; 87: 1086–1093. 16761585 10.1890/0012-9658(2006)87[1086:roptla]2.0.co;2

[11] Gorini L, Linnell JDC, May R, Panzacchi M, Boitani L, Odden M, et al Habitat heterogeneity and mammalian predator–prey interactions. Mamm Rev. 2012; 42: 55–77.

[12] Dupke C, Bonenfant C, Reineking B, Hable R, Zeppenfeld T, Ewald M, et al Habitat selection by a large herbivore at multiple spatial and temporal scales is primarily governed by food resources. Ecography. 2016; doi: 10.1111/ecog.02152

[13] Werner EE, Gilliam JF, Hall DJ, Mittelbach GG. An Experimental Test of the Effects of Predation Risk on Habitat Use in Fish. Ecology. 1983; 64: 1540–1548.

[14] Jordan F, Bartolini M, Nelson C, Patterson PE, Soulen HL. Risk of predation affects habitat selection by the pinfish Lagodon rhomboides (Linnaeus). J Exp Mar Bio Ecol. 1997; 208: 45–56.

[15] Evans KL. The potential for interactions between predation and habitat change to cause population declines of farmland birds. Ibis. 2004; 146: 1–13.

[16] Doran PJ, Holmes RT. Habitat occupancy patterns of a forest dwelling songbird: causes and consequences. Can J Zool. 2005; 83: 1297–1305.

[17] Morosinotto C, Villers A, Thomson RL, Varjonen R, Korpimäki E. Competitors and predators alter settlement patterns and reproductive success of an intraguild prey. Ecol Monog. 2017; 87: 4–21.

[18] Dickman CR. Predation and Habitat Shift in the House Mouse, Mus Domesticus. Ecology. 1992; 73: 313–322.

[19] Barreto GR, Rushton SP, Strachan R, Mcdonald DW. The role of habitat and mink predation in determining the status and distribution of water voles in England. Anim Conserv. 1998; 1: 129–137.

[20] Widén P. Goshawk predation during winter, spring and summer in a boreal forest area of Central Sweden. Holar Ecol. 1987; 10: 104–109.

[21] Korpimäki E, Huhtala K, Sulkava S. Does the Year-to-Year Variation in the Diet of Eagle and Ural Owls Support the Alternative Prey Hypothesis? Oikos. 1990; 58:47–54.

[22] Carey AB, Horton SP, Biswell BL. Northern spotted owls: influence of prey base and landscape character. Ecol Monogr. 1992; 62: 223–250.

[23] Tornberg R, Korpimäki E, Byholm P. Ecology of the Northern Goshawk in Fennoscandia. Stud Av Biol. 2006; 31:141–157.

[24] Steele M. Evolutionary interactions between tree squirrels and trees: a review and synthesis. Curr Sci. 2008; 95: 871–876.

[25] Hurme E, Mönkkönen M, Reunanen P, Nikula A, Nivala V. Temporal patch occupancy dynamics of the Siberian flying squirrel in a boreal forest landscape. Oikos 2008; 31: 469–476.

[26] Nupp TE, Swihart RK. Landscape-Level Correlates of Small-Mammal Assemblages in Forest Fragments of Farmland. J Mammal. 2000; 81:512–526.

[27] Selonen V, Sulkava P, Sulkava R, Korpimäki E. Decline of flying and red squirrels in boreal forests revealed by long-term diet analyses of avian predators. Anim Conserv. 2010; 13: 579–585.

[28] Koskimäki J, Huitu O, Kotiaho JS, Lampila S, Mäkelä A, Sulkava R, et al Are habitat loss, predation risk and climate related to the drastic decline in a Siberian flying squirrel population? A 15-year study. Population Ecology. 2014; 56: 341–348.

[29] Rajala P, Lampio T. Oravan ravinnosta maassamme vuonna 1945–1961 (In Finnish with English summary: Food of the squirrel (*Sciurus vulgaris*) in Finland in 1945–1961). Suomen Riista. 1963; 16:155–185.

[30] Selonen V, Mäkeläinen S. Ecology and protection of a flagship species, the Siberian flying squirrel. Hystrix. 2017; 28: 2.

[31] Hokkanen H, Törmälä T, Vuorinen H. Decline of the flying squirrel Pteromys volans l. populations in Finland. Biol Conserv. 1982; 23: 273–284.

[32] Hanski IK, Henttonen H, Liukko U-M, Meriluoto M, Mäkelä A. Liito-oravan (Pteromys volans) biologia ja suojelu Suomessa Suomen ympäristö 459, Ympäristöministeriö, Helsinki, 2001 (in Finnish with Swedish abstract).

[33] Lampila S, Wistbacka R, Mäkelä A, Orell M. Survival and population growth rate of the threatened Siberian flying squirrel (*Pteromys volans*) in a fragmented forest landscape. Ecoscience. 2009; 16:66–74.

[34] Rassi P, Hyvärinen E, Juslén A, Mannerkoski I, editors. The 2010 Red List of Finnish Species. Ympäristöministeriö & Suomen ympäristökeskus, Helsinki; 2010.

[35] Liukko U-M, Henttonen H, Hanski IK, Kauhala K, Kojola I, Kyheröinen E-M, et al Suomen nisäkkäiden uhanalaisuus 2015 –The 2015 Red List of Finnish Mammal Species. Ympäristöministeriö & Suomen ympäristökeskus; 2016.

[36] Mikkola H. Owls of Europe. London: AD and T Poyser; 1983.

[37] Byholm P, Burgas D, Virtanen T, Valkama J. Competitive exclusion within the predator community influences the distribution of a threatened prey species. Ecology 2012; 93: 1802–1808. 22928409 10.1890/12-0285.1

[38] Korpimäki E. Niche relationships and life history tactics of three sympatric Strix owl species in Finland. Ornis Scandinavica. 1986; 17: 126–132.

[39] Hanski IK. Home ranges and habitat use in the declining flying squirrel, *Pteromys volans*, in managed forests. Wildlife Biol. 1998; 4:33–46.

[40] Turkia T, Selonen V, Danilov P, Kurhinen J, Ovaskainen O, Rintala J, et al Red squirrels decline in abundance in boreal forests of Finland and NW Russia. Ecography. 2018; 40: 001–009.

[41] Andrén H, Lemnell P-A. Population fluctuations and habitat selection in the Eurasian red squirrel Sciurus vulgaris. Ecography. 1992; 15: 303–307.

[42] Selonen V, Varjonen R, Korpimäki E. Immediate or lagged responses of a red squirrel population to pulsed resources. Oecologia. 2015; 177: 401–411. doi: 10.1007/s00442-014-3148-7 25413865 10.1007/s00442-014-3148-7

[43] Moller H. Foods and foraging behavior of red (Sciurus vulgaris) and grey (Sciurus carolinensis) squirrels. Mamm Rev. 1983; 13: 81–98.

[44] Wauters LA, Gurnell J, Preatoni D, Tosi G. Effects of spatial variation in food availability on spacing behaviour and demography of Eurasian red squirrels. Ecography. 2001; 24: 525–538.

[45] Gurnell J. Squirrel numbers and the abundance of tree seeds. Mamm Rev. 1983; 13: 133–148.

[46] Hakkarainen H, Mykrä S, Kurki S, Korpimäki E, Nikula A, Koivunen V. Habitat composition as a determinant of reproductive success of Tengmalm's owls under fluctuating food conditions. Oikos. 2003; 100:162–171.

[47] Selonen V, Hanski IK. Dispersing Siberian flying squirrels (Pteromys volans) locate preferred habitats in fragmented landscapes. Can J Zool. 2012; 90: 885–892.

[48] Korpimäki E, Hakkarainen H. The boreal owl: ecology, behaviour and conservation of a forest-dwelling predator. Cambridge University Press; 2012.

[49] R Core Team. R: A language and environment for statistical computing. R Foundation for Statistical Computing, Vienna, Austria; 2016.

[50] Andrén H, Delin A. Habitat Selection in the Eurasian Red Squirrel, Sciurus vulgaris, in Relation to Forest Fragmentation. Oikos. 1994; 70: 43–48.

[51] Hanski IK, Stevens PC, Ihalempiä P, Selonen V. Home-Range Size, Movements, and Nest-Site Use in the Siberian Flying Squirrel, Pteromys Volans. J Mammal. 2000; 81: 798–809.

[52] Selonen V, Painter JN, Rantala S, Hanski IK. Mating system and reproductive success in the Siberian flying squirrel. J Mammal. 2013; 94:1266–1273.

[53] Selonen V, Hanski IK. Occurrence and dispersal of the red squirrel and the Siberian flying squirrel in fragmented landscapes In: Shuttleworth CM, Lurz PWW, Hayward MW, editors. Red squirrels: ecology, conservation & management in Europe. Woodbridge, Suffolk, European Squirrel Initiative: 2015; pp. 67–82.

[54] Mikkola A, Jaakkola O, Sucksdorff Y. The Slices-project: National classification of land use, land cover and soil, and the production of databases. The Finnish Environment. 1999; 342: 1–86.

[55] Vuerola I. Satellite Image Based Land Cover and Forest Classification of Finland. In: Kuittinen R, editor. Proceedings of the Finnish-Russian seminar on remote sensing in Helsinki, 29th August–1st September, 1994. (Reports of the Finnish Geodetic Institue 97: 2). Masal, Finland: Finnish Geodetic Institute; 1997. Pp. 42–52.

[56] METLA. Forest classification open data. http://kartta.metla.fi/ Luonnonvarakeskus, 2012.

[57] Saurola P. Mate and nest-site fidelity in Ural and tawny owls. In: Nero RW, Clark RJ, Knapton RJ, Hamre RH, editors. Biology and conservation of northern forest owls, Symposium proceedings. Manitoba (Canada): USDA Forest Service General Techical Report; 1987. pp. 81–86.

[58] Selonen V, Varjonen R, Korpimäki E. Predator presence, but not food supplementation, affects forest red squirrels in winter. Ann Zool Fennici. 2016; 53: 183–193.

[59] Roth TC II, Lima SL. Use of Prey Hotspots by an Avian Predator: Purposeful Unpredictability? Am Nat. 2007; 169: 264–273. doi: 10.1086/510605 17211809 10.1086/510605

[60] Roth TC II, Vetter WE, Lima SL. Spatial Ecology of Winting Accipiter Hawks: Home Range, Habitat Use, and the Influence of Bird Feeders. Condor. 2008; 110: 260–268.

[61] Björklund H, Santangeli AF, Blanchet G, Huitu O, Lehtoranta H, Lindén H, et al Intraguild predation and competition impacts on a subordinate predator. Oecologia. 2016; 181: 257–269. doi: 10.1007/s00442-015-3523-z 26841931 10.1007/s00442-015-3523-z

[62] Hokkanen T. Seed crops and seed crop forecasts for a number of tree species. Finn For Res Inst Res Pap. 2000; 790: 87–97.

[63] Zamorano JG, Hokkanen T, Lehikoinen A. Climate-driven synchrony in seed production of masting deciduous and conifer tree species. J Plant Ecol. 2016; doi: 10.1093/jpe/rtw117

[64] Bates D, Maechler M, Bolker B, Walker S. Fitting Linear Mixed-Effects Models Using lme4. J Stat Softw. 2015; 67: 1–48.

[65] Schielzeth H. Simple means to improve the interpretability of regression coefficients. Methods Ecol Evol. 2010; 1: 103–113.

[66] Santangeli A, Hanski IK, Mäkelä H. Integrating multi-source forest inventory and animal survey data to assess nationwide distribution and habitat correlates of the Siberian flying squirrel. Biol Conserv. 2013; 157: 31–38.

[67] Fox J, Weisberg S. An {R} Companion to Applied Regression, Second Edition Thousand Oaks CA: Sage; 2011.

[68] Neter J, Wasserman W, Kutner MH. Applied Linear Regression Models. Homewood, IL: Irwin; 1989.

[69] O’Brien RM. A Caution Regarding Rules of Thumb for Variance Inflation Factors. Qual Quant. 2007; 41: 673–690.

[70] Calcagno V. glmulti: Model selection and multimodel inference made easy. R package version 1.0.7 2013 https://CRAN.R-project.org/package=glmulti

[71] Paradis E, Claude J, Strimmer K. APE: analyses of phylogenetics and evolution in R language. Bioinformatics. 2004; 20: 289–290. 14734327 10.1093/bioinformatics/btg412

[72] Forsman ED, Meslow EC, Wight HM. Distribution and biology of the spotted owl in Oregon. Wildlife Monographs. 1984; 98: 1–64.

[73] Pietiäinen H. Seasonal and individual variation in the production of offspring in the Ural Owl Strix uralensis. J Anim Ecol. 1989; 58: 905–920.

[74] Hakkarainen H, Korpimäki E. Competitive and predatory interactions among raptors: an observational and experimental study. Ecology. 1996; 77: 1134–1142.

[75] Rettie WJ, Messier F. Hierarchical Habitat Selection by Woodland Caribou: Its Relationship to Limiting Factors. Ecography. 2000; 23: 466–478.

[76] Dussault C, Ouellet J-P, Courtois R Hout J, Breton L, Jolicoeur H. Linking moose habitat selection to limiting factors. Ecography. 2005; 28: 619–628.

[77] Valeix M, Loveridge AJ, Chamaillé-Jammes S, Davidson Z, Murindagomo F, Fritz H, et al Behavioral adjustments of African herbivores to predation risk by lions: Spatiotemporal variations influence habitat use. Ecology. 2009; 90: 23–30. 19294909 10.1890/08-0606.1

[78] Doncaster CP. Factors Regulating Local Variations in Abundance: Field Tests on Hedgehogs, Erinaceus europaeus. Oikos. 1994; 69: 182–192.

[79] Creel S, Winnie J Jr, Maxwell B, Hamlin K, Creel M. Elk alter habitat selection as an antipredator response to wolves. Ecology. 2005; 86: 3387–3397.

[80] Hoset KS, Koivisto E, Huitu O, Ylönen H, Korpimäki E. Multiple predators induce risk reduction in coexisting vole species. Oikos. 2009; 118: 1421–1429.

[81] Petty SJ, Lurz PWW, Rushton SP. Predation of red squirrels by northern goshawks in a conifer forest in northern England: can this limit squirrel numbers and create a conservation dilemma? Biol Conserv. 2003; 111: 105–114.

[82] Delin AE, Andrén H. Effects of habitat fragmentation on Eurasian red squirrel (Sciurus vulgaris) in a forest landscape. Landsc Ecol. 1999: 14: 67.

[83] Selonen V, Hanski IK, Stevens P. Space use of the Siberian flying squirrel Ptevomys volans in fragmented forest landscapes. Ecography. 2001; 24: 588–600.

[84] Magris L, Gurnell J. Population ecology of the red squirrel (Sciurus vulgaris) in a fragmented woodland ecosystem on the Island of Jersey, Channel Islands. J Zool. 2002; 256: 99–112.

[85] Hoset KS, Villers A, Wistbacka R, Selonen V. Pulsed food resources, but not forest cover, determine lifetime reproductive success in a forest-dwelling rodent. J Anim Ecol. 2017; 86: 1235–1245. doi: 10.1111/1365-2656.12715 28636171 10.1111/1365-2656.12715

[86] Wheatley M, Fisher JT, Larsen K, Litke J, Boutin S. Using GIS to relate small mammal abundance and landscape structure at multiple spatial extents: the northern flying squirrel in Alberta, Canada. J Appl Ecol. 2005; 42: 577–586.

[87] Trudeau C, Imbeau L, Drapeau P, Mazerolle MJ. Site occupancy and cavity use by the northern flying squirrel in the boreal forest. J Wildl Manage. 2011; 75: 1646–1656.

[88] Ritchie LE, Betts MG, Forbes G, Vernes K. Effects of landscape composition and configuration on northern flying squirrels in a forest mosaic. For Ecol Manage. 2009; 257: 1920–1929.

[89] Selonen V, Wistbacka R. Siberian flying squirrels do not anticipate future resource abundance. BMC Ecol. 2016; 16: 51 doi: 10.1186/s12898-016-0107-7 27842537 10.1186/s12898-016-0107-7 PMC5109687

[90] Jokimäki J, Selonen V, Lehikoinen A, Kaisanlahti-Jokimäki M-L. The role of urban habitats in the abundance of red squirrels (*Sciurus vulgaris*, L.) in Finland. Urban For Urban Green. 2017; 27: 100–108.

[91] Sullivan TP. Responses of Red Squirrel (Tamiasciurus hudsonicus) populations to supplemental food. J Mammal. 1990; 71: 579–590.

[92] Klenner W, Crebs CJ. Red Squirrel Population Dynamics I. The Effect of Supplemental Food on Demography. J Anim Ecol. 1991; 60: 961–978.

[93] Lurz PWW, Garson PJ, Wauters LA. Effects of temporal and spatial variations in food supply on the space and habitat use of red squirrels (Sciurus vulgaris L.). J Zool. 2000; 251: 167–178.

[94] Di Pierro E, Ghisla A, Wauters LA, Molinari A, Martinoli A, Gurnell J, et al The effects of seed availability on habitat use by a specialist seed predator. Eur J Wild Res. 2011; 57: 585–595.

[95] Santangeli A, Wistbacka R, Hanski IK, Laaksonen T. Ineffective enforced legislation for nature conservation: A case study with Siberian flying squirrel and forestry in a boreal landscape. Biol Conserv. 2013; 157: 237–244.

[96] Jokinen M, Mäkeläinen S, Ovaskainen O. ‘Strict’, yet ineffective: legal protection of breeding sites and resting places fails with the Siberian flying squirrel. Anim Conserv. 2015; 18: 167–175.

[97] Melin M, Mehtätalo L, Miettinen J, Tossavainen S, Packalen P. Forest structure as a determinant of grouse brood occurrence—An analysis linking LiDAR data with presence/absence field data. For Ecol Manage. 2016; 380: 202–211.

