# Supplementary material for: Predation risk landscape modifies flying and red squirrel nest site occupancy independently of habitat amount
Source: PLoS One. 2018 Mar 29;13(3):e0194624. doi: 10.1371/journal.pone.0194624 (PMC5875771; doi:10.1371/journal.pone.0194624)
Supplement: S1 Table — (DOCX) [file pone.0194624.s001.docx]

Supporting Information for “**Predation risk landscape modifies flying and red squirrel nest site occupancy independently of habitat amount**” by Tytti Turkia, Erkki Korpimäki, Alexandre Villers and Vesa Selonen.

# Supporting Information.

**S1 Table. Areas of different habitat types within small and large buffers around occupied (O) and unoccupied (U) squirrels nest boxes.** Buffer sizes are 200 m and 1 km for flying squirrel nest boxes and 300 m and 2.5 km for red squirrel nest boxes. See Table 1 in the research article for abbreviations of habitat types. Values are means±SD in hectares.

|  | Flying squirrel | |  |  | Red Squirrel | |  |  |
| --- | --- | --- | --- | --- | --- | --- | --- | --- |
|  | O | U | O | U | O | U | O | U |
|  | small | small | large | large | small | small | large | large |
| Clear cut | 2.2±1.5 | 2.3±1.6 | 47.2±25.1 | 53.2±27.2 | 4.3±2.8 | 4.4±2.9 | 322.5±116.9 | 317.8±113.5 |
| Young pine | 1.5±0.9 | 2±1.2 | 39.3±23.5 | 50.7±28.3 | 3.3±2.1 | 3.8±2.4 | 274.8±110.4 | 303.1±116.7 |
| Young birch | 0.5±0.6 | 0.5±0.6 | 9.7±5.8 | 10.9±7.7 | 1.1±1 | 1.1±1 | 64.4±27.3 | 66.1±29.9 |
| Young mixed | 0.1±0.2 | 0.1±0.1 | 2±1.5 | 2.2±1.6 | 0.2±0.2 | 0.2±0.2 | 14.1±6.6 | 14.4±6.7 |
| Mo  birch | 0.1±0.1 | 0.1±0.2 | 1.6±1.6 | 1.8±1.8 | 0.1±0.3 | 0.2±0.3 | 9.3±6 | 10.1±6.7 |
| Mo  pine | 3.2±2 | 3.5±1.9 | 60.7±33 | 67.9±33.9 | 6±3.7 | 6.5±3.8 | 396.6±128 | 421.7±130.6 |
| Mo spruce | 0.4±0.6 | 0.3±0.6 | 3.6±4.3 | 3.6±3.8 | 0.3±0.7 | 0.4±0.8 | 17±12.6 | 20.3±13.7 |
| Mo birch pine | 0.3±0.4 | 0.3±0.4 | 6.5±4.6 | 7.5±4.7 | 0.5±0.6 | 0.6±0.6 | 39.3±17.9 | 43.2±18.7 |
| Mo pine spruce | 0.9±0.9 | 0.8±0.8 | 10.6±8.3 | 10.9±8.2 | 1.2±1.2 | 1.3±1.4 | 65.6±34.1 | 66.4±36.8 |
| Mo birch spruce | 0±0.1 | 0±0.1 | 1±1 | 1±1.1 | 0.1±0.2 | 0.1±0.2 | 4.5±3.1 | 5.3±3.4 |
| Built | 0.6±0.4 | 0.5±0.4 | 8.2±6.6 | 7.5±6.3 | 1.1±0.8 | 1±0.8 | 61.6±32.4 | 56.6±33.3 |
| Bog | 0.3±0.4 | 0.3±0.6 | 9.9±14.9 | 13.9±16.6 | 0.7±0.8 | 0.8±1.3 | 94.7±84.8 | 103.3±83.6 |
| Field | 2.2±2.3 | 1.5±2.2 | 75.5±58.3 | 48.5±52.8 | 8.7±6.1 | 6.9±6.1 | 529±301.3 | 458.8±289.2 |
