# Supplementary material for: Predation risk landscape modifies flying and red squirrel nest site occupancy independently of habitat amount
Source: PLoS One. 2018 Mar 29;13(3):e0194624. doi: 10.1371/journal.pone.0194624 (PMC5875771; doi:10.1371/journal.pone.0194624)
Supplement: S2 Table — (DOCX) [file pone.0194624.s002.docx]

Supporting Information for “**Predation risk landscape modifies flying and red squirrel nest site occupancy independently of habitat amount**” by Tytti Turkia, Erkki Korpimäki, Alexandre Villers and Vesa Selonen.

# Supporting Information

**S2 Table. Yearly occupancy rates of flying and red squirrel nest boxes.**

|  | Flying squirrel | | | | Red squirrel | | | |
| --- | --- | --- | --- | --- | --- | --- | --- | --- |
| Year | Occupied | Empty | Total | %Occupied | Occupied | Empty | Total | %Occupied |
| 1999 |  |  |  |  | 46 | 346 | 392 | 11.7 |
| 2000 |  |  |  |  | 40 | 382 | 422 | 9.5 |
| 2001 |  |  |  |  | 85 | 310 | 395 | 21.5 |
| 2002 | 8 | 91 | 99 | 8.1 | 30 | 345 | 375 | 8 |
| 2003 | 15 | 89 | 104 | 14.4 | 34 | 346 | 380 | 8.9 |
| 2004 | 7 | 231 | 238 | 2.9 | 37 | 344 | 381 | 9.7 |
| 2005 | 22 | 284 | 306 | 7.2 | 54 | 357 | 411 | 13.1 |
| 2006 | 28 | 297 | 325 | 8.6 | 76 | 324 | 400 | 19 |
| 2007 | 34 | 332 | 366 | 9.3 | 141 | 264 | 405 | 34.8 |
| 2008 | 39 | 350 | 389 | 10.0 | 153 | 253 | 406 | 37.7 |
| 2009 | 33 | 400 | 433 | 7.6 | 117 | 300 | 417 | 28.1 |
| 2010 | 38 | 420 | 458 | 8.3 | 107 | 320 | 427 | 25.1 |
| 2011 | 59 | 409 | 468 | 12.6 | 85 | 348 | 433 | 19.6 |
| 2012 | 52 | 421 | 473 | 11.0 | 111 | 362 | 473 | 23.5 |
| 2013 | 54 | 409 | 463 | 11.7 | 129 | 347 | 476 | 27.1 |
| 2014 | 51 | 409 | 460 | 11.1 | 109 | 318 | 427 | 25.5 |
| 2015 | 53 | 361 | 414 | 12.8 | 84 | 348 | 432 | 19.4 |
