# Supplementary material for: Predation risk landscape modifies flying and red squirrel nest site occupancy independently of habitat amount
Source: PLoS One. 2018 Mar 29;13(3):e0194624. doi: 10.1371/journal.pone.0194624 (PMC5875771; doi:10.1371/journal.pone.0194624)
Supplement: S3 Table — (DOCX) [file pone.0194624.s003.docx]

Supporting Information for “**Predation risk landscape modifies flying and red squirrel nest site occupancy independently of habitat amount**” by Tytti Turkia, Erkki Korpimäki, Alexandre Villers and Vesa Selonen.

# Supporting Information

**S3 Table. Alternative models.** The 10 best models for a) flying squirrel occurrence in nest boxes using small buffers (200 m) and b) large buffers (1 km) for landscape variables, c) red squirrel occurrence in nest boxes using small buffers (300 m) and d) large buffers (2500 m) for landscape variables. Note that several models may have same AIC value, i.e. equally much support. Models within 2 AIC units from the lowest AIC are considered equally good. These are indicated with an *. All models include a random factor as described in methods (+(1 |SITEID/BCODE) for flying squirrel models and +(1 |SITEID) for red squirrels), which is not shown in formula for the sake of simplicity. All models were fitted with function ‘glmer’ from package ‘lme4’ and had a binomial distribution. Model selection was done with function ‘glmulti’ (package ‘glmulti’) using a genetic algorithm which looked for 100 models. Variables with significant effect (*P* < 0.05) are printed in bold. The directions of the effects (positive or negative) are as in full models, (i.e. negative for the Ural owl, for example, see Tables 2 and 3), expect for the effect of Goshawk^2^, which is positive when Goshawk is included in the model and negative without it.

a)

|  | AIC | Model |
| --- | --- | --- |
| 1* | 2640.2 | ptevol ~ 1 + **Year**+ **Ural_owl**+ **Field**+ **Field^2^** + **Goshawk** |
| 2* | 2641.8 | ptevol ~ 1 + **Year**+ **Ural_owl**+ **Field**+ **Field^2^**+ Goshawk + Goshawk^2^ |
| 3 | 2644.8 | ptevol ~ 1 + **Year**+ Y mix + **Ural_owl**+ **Field**+ **Goshawk** |
| 4 | 2645.5 | ptevol ~ 1 + **Year**+ **Ural_owl**+ **Field** + **Goshawk** |
| 5 | 2645.6 | ptevol ~ **1** + **Year**+ **ym** + **Ural_owl**+ **Field**+ **Field^2^** |
| 6 | 2645.7 | ptevol ~ **1** + **Year**+ Y mix + **Ural_owl**+ **Field**+ **Goshawk^2^** |
| 7 | 2646.7 | ptevol ~ 1 + **Year**+ Y mix + **Ural_owl**+ **Field**+ Goshawk + Goshawk^2^ |
| 8 | 2646.9 | ptevol ~ **1** + **Year**+ **Ural_owl**+ **Field**+ **Goshawk^2^** |
| 9 | 2647.3 | ptevol ~ 1 + **Year**+ **Ural_owl**+ **Field**+ Goshawk + Goshawk^2^ |
| 10 | 2649.3 | ptevol ~ **1** + **Year**+ **Y mix** + **Ural_owl**+ **Field** |

b)

|  | AIC | Model |
| --- | --- | --- |
| 1* | 2625.0 | ptevol ~ 1 + **Year**+ **Built** + **Ural_owl**+ **Field** + **Goshawk** |
| 2* | 2625.7 | ptevol ~ 1 + **Year**+ **Built** + **Ural_owl**+ **Field** + Field^2^ + **Goshawk** |
| 3* | 2626.3 | ptevol ~ 1 + **Year**+ **Built** + **Ural_owl**+ **Field** + Goshawk + Goshawk^2^ |
| 4* | 2626.9 | ptevol ~ **1** + **Year**+ **Built** + **Ural_owl**+ **Field** + **Goshawk^2^** |
| 5* | 2626.9 | ptevol ~ 1 + **Year**+ **Built** + **Ural_owl+ Field** + Field^2^ + Goshawk + Goshawk^2^ |
| 6 | 2627.6 | ptevol ~ **1** + **Year**+ **Built** + **Ural_owl**+ **Field** + Field^2^ + **Goshawk^2^** |
| 7 | 2630.8 | ptevol ~ **1** + **Year**+ **Built** + **Ural_owl**+ **Field** |
| 8 | 2631.8 | ptevol ~ **1** + **Year**+ **Built** + **Ural_owl**+ **Field** + Field^2^ |
| 9 | 2632.1 | ptevol ~ 1 + **Year**+ Mo birch + **Ural_owl**+ **Field** + **Goshawk** |
| 10 | 2633.9 | ptevol ~ 1 + **Year**+ **Ural_owl**+ **Field** + **Goshawk** |

c)

|  | AIC | Model |
| --- | --- | --- |
| 1* | 6703.1 | red ~ **1** + **TimePeriod2** + **Year** + **Built** + Bog + **Field** + **Field^2^** + Goshawk |
| 2* | 6703.2 | red ~ **1** + **TimePeriod2** + **Year** + Mo birch pine + **Built** + **Field** + **Field^2^** + Goshawk |
| 3* | 6703.7 | red ~ **1** + **TimePeriod2** + **Year** + Mo birch pine + **Built** + **Field** + **Field^2^** + Goshawk |
| 4* | 6704.2 | red ~ **1** + **TimePeriod2** + **Year** + Mo birch pine + **Built** + **Field** + **Field^2^** |
| 5* | 6704.7 | red ~ **1** + **TimePeriod2** + **Year** + Mo spruce + **Built** + **Field** + **Field^2^** + Goshawk |
| 6* | 6705.0 | red ~ **1** + **TimePeriod2** + **Year** + **Built** + **Field** + **Field^2^** + Goshawk |
| 7* | 6705.1 | red ~ **1** + **TimePeriod2** + **Year** + Mo birch + **Built** + **Field** + **Field^2^** + Goshawk |
| 8 | 6705.3 | red ~ **1** + **TimePeriod2** + **Year** + **Built** + **Field** + **Field^2^** |
| 9 | 6709.2 | red ~ **1** + **TimePeriod2** + **Built** + **Field** + **Field^2^** + Goshawk |
| 10 | 6709.4 | red ~ **1** + **TimePeriod2** + **Year** + **Built** + **Field** + Goshawk |

d)

|  | AIC2 | Model |
| --- | --- | --- |
| 1* | 6714.0 | red ~ **1** + **TimePeriod2** + **Year** + **Y mix** + **Mo birch spruce** + **Field** + **Field^2^** + **Goshawk** |
| 2 | 6716.2 | red ~ **1** + **TimePeriod2** + **Year** + **ym** + **Mo birch spruce** + **Field** + **Goshawk** |
| 3 | 6717.1 | red ~ **1** + **TimePeriod2** + **Year** + clear cut + **Mo birch spruce** + **Field** + Field^2^ + **Goshawk** |
| 4 | 6717.5 | red ~ **1** + **TimePeriod2** + **Year** + **Mo birch spruce** + **Field** + **Field^2^** + **Goshawk** |
| 5 | 6717.9 | red ~ **1** + **TimePeriod2** + **Year** + **Y mix** + **Mo birch spruce** + **Field** + **Field^2^** |
| 6 | 6720.6 | red ~ **1** + **TimePeriod2** + **Year** + **Y mix** + **Mo birch spruce** + **Field** |
| 7 | 6720.6 | red ~ **1** + **TimePeriod2** + **Year** + **Mo birch spruce** + Built + **Field** + **Field^2^** |
| 8 | 6721.1 | red ~ **1** + **TimePeriod2** + **Year** + **Mo birch spruce** + **Field** + **Field^2^** |
| 9 | 6722.2 | red ~ **1** + **TimePeriod2** + **Year** + **Mo birch spruce** + **Field** + **Goshawk** |
| 10 | 6723.8 | red ~ **1** + **TimePeriod2** + **Year** + **Mo birch spruce** + **Built** + **Field** |
